# Meta-analysis identifies candidate key genes in endometrium as predictive biomarkers for clinical pregnancy in IVF

**DOI:** 10.18632/oncotarget.22096

**Published:** 2017-10-26

**Authors:** Jingyu Li, Dongyun Liu, Jiang Wang, Huali Deng, Xiu Luo, Xiaoli Shen, Yanjun Huan, Guoning Huang, Hong Ye

**Affiliations:** ^1^ Chongqing Reproductive and Genetics Institute, Yu Zhong District, Chongqing, China; ^2^ College of Veterinary Medicine, Qingdao Agricultural University, Qingdao, China

**Keywords:** implantation failure, endometrial receptivity, microarray, meta-analysis, IVF

## Abstract

Genetic factors in endometrium are likely to be involved in the embryo implantation failure (IF), one of the major limiting factors in the success of *in vitro* fertilization (IVF). In this study, we aimed to identify critical genes from the transcriptional profile for the establishment of the endometrial receptivity which supporting the normal pregnancy. Three GEO datasets, including 12 samples of IF and 12 samples of controls, were used for the meta-analysis. We identified 182 different expression genes (DEGs) by comparing IF with controls and present here the successful clustering according to sample type, not by the origin. The gene ontology (GO) enriched analysis demonstrated the significant downregulation in activation and regulation of inflammatory and immune response in IF patients. Furthermore, network analysis of down-regulated genes identified the significant hub genes containing *GADD45A* (growth arrest and DNA damage inducible alpha, Degree = 77), *GZMB* (granzyme B, Degree = 38) and *NLRP2* (NLR family pyrin domain containing 2, Degree = 37). The lower expression of *NLRP2*, related to inflammatory responses with the most degree in the network, was validatied by other GEO data. Besides, it was confirmed that the *NLRP2* could act as a predictor for pregnancy after IVF (AUC = 87.93%; sensitivity, 60.00%; specificity, 91.30% ). Our meta-analysis will help us to better understand the molecular regulation of endometrial receptivity, and guiding further line of treatment for IF during IVF.

## INTRODUCTION

Despite of advancement in the assisted reproductive techniques (ART), current pregnancy and live birth success rates still remain unsatisfactory [1, 2]. The major reason for this limited success is embryo implantation failure (IF), which is mainly caused by the low-quality embryo and impaired endometrial receptivity [3]. The embryo quality has been the most focused player in this process, with many decades of embryological research [4–7]. However, a significant proportion of couples still undergoing IVF experience IF, even after the good-quality embryo transfer. Endometrial dysfunction could be one of the major factors related to the IF in this condition [3]. Therefore, elucidation of the molecular mechanisms controlling endometrial receptivity is important for improving the success rate of ART.

Embryo implantation can only occur during a temporally restricted period from day 20 to day 24 of the menstrual cycle, called the window of implantation (WOI) [8]. The endometrium is nonreceptive in the most of the menstrual cycle, prohibiting the adherence and implantation proceeding. During the WOI, however, the endometrium is receptive and permits a blastocyst to attach and invade. Previous studies have indicated the important roles of inflammatory and immune response in embryo implantation, through allowing the invasion and maintenance of semi-allogenic embryo in the endometrium [9, 10]. In addition, several biological processes including cell proliferation [11, 12], cytokines and estradiol response have been considered to be responsible for successful implantation [11, 13, 14]. Thus, the precise regulation of gene expression during WOI is required for the establishment of endometrial receptivity.

High-throughput technology is useful for monitoring the transcriptomes of endometrium during the WOI. Recent studies have enabled advances in understanding the molecular mechanisms of endometrial receptivity and demonstrated the feasibility of the diagnosis of endometrial receptivity status with the use of transcriptomic profiling [12, 15–19]. Although many long lists of key gene signatures of endometrial receptivity were identified, there tends to be inconsistencies among studies due to the differences in experimental designs, microarray type, day of the cycle when the sample is collected, and other reasons [12, 15–19]. To address these challenges, meta-analysis has been applied for large-scale comparative analysis of multiple gene expression datasets, which can enhance statistical power in identifying more robust and reliable gene signatures [20]. However, similar meta-analysis has never been conducted for IF.

In this study, we performed the first meta-analysis of endometrial gene expression datasets from various IF studies to overcome the limitation of heterogeneity. We focused our main attention on genes that are down-regulated, since a strong skew towards down-regulation of processes in IF patients was detected through Gene Ontology (GO) enrichment analysis. Network analysis identified *NLPR2* (NLR family pyrin domain containing 2), as the most important hub gene related to inflammatory response. Moreover, receiver operating characteristic (ROC) analysis of *NLRP2* suggested its potential to be a single predictive biomaker for clinical pregnancy in IVF. These findings deepened the understanding of the molecular mechanisms of endometrial receptivity, and identifying a novel list of candidate key genes associated with IF.

## RESULTS

### Studies included in the meta-analysis

**Figure 1:**
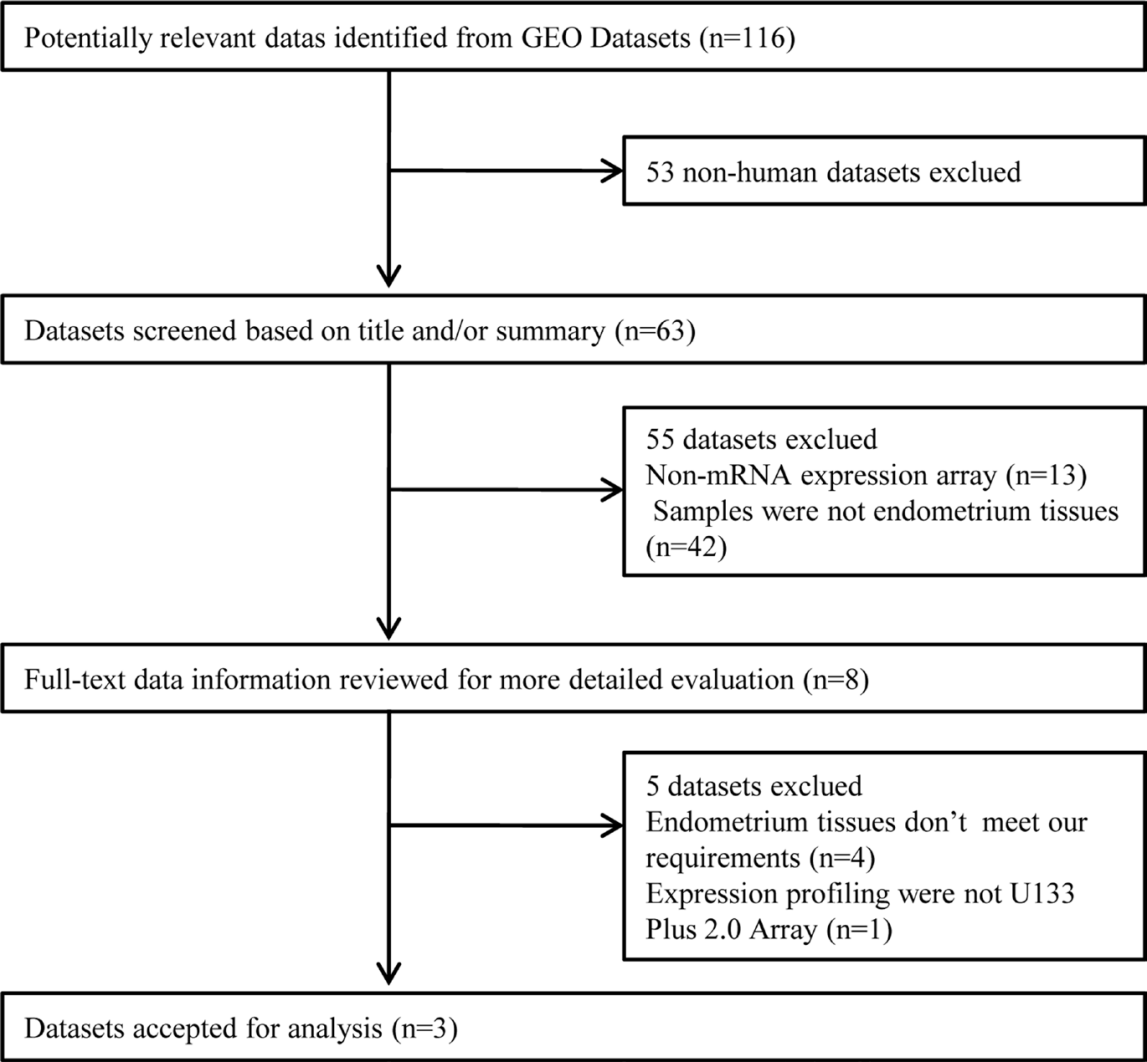
Flowchart of the selected process of microarray datasets for the meta-analysis

A total of 116 datasets were identified by electronic search, 108 of which were excluded because 53 were animal studies, 13 were not mRNA expression array, and 42 didn't contained endometrial samples. Thus, 8 microarray datasets were selected for full-text data review for more detailed evaluation. Five datasets were excluded because 1 microarray arary platform didn't meet the requirement, and the endometrial samples in the other 4 microarray datasets were not from IF and control patients in IVF cycle. Finally, three microarray datasets met the inclusion criteria and were considered for subsequent analysis. The flowchart outlining the selection process in detail is shown in Figure 1. The details of the individual microarray dataset analyzed in this study are summarized in Table 1.

**Table 1:** Microarray studies in endometrium used for analysis

GEO accession no.	No. of samples	Platform
GSE18140	Pregnant = 4; Nonpregnant = 4	GPL570 [HG-U133_Plus_2] AffymetrixHuman Genome U133 Plus 2.0 Array
GSE21225	Pregnant = 3; Nonpregnant = 3	GPL570 [HG-U133_Plus_2] AffymetrixHuman Genome U133 Plus 2.0 Array
GSE26787	Pregnant = 5; Nonpregnant = 5	GPL570 [HG-U133_Plus_2] AffymetrixHuman Genome U133 Plus 2.0 Array

### Meta-analysis of endometrial gene expression patterns in control vs. IF patient

**Figure 2:**
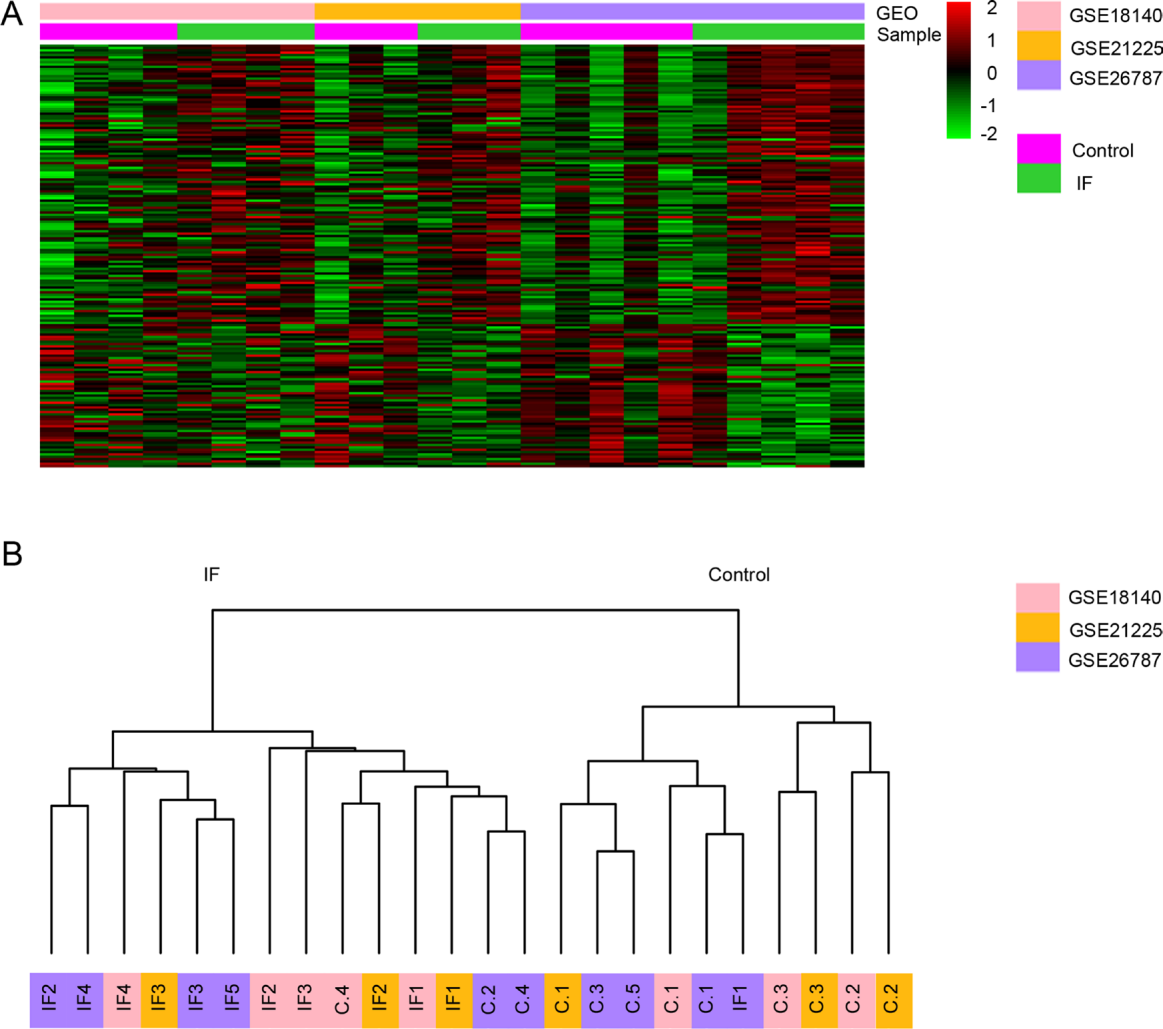
Genes differentially expressed in endometrium between IF and control patients across three datasets (**A**) Heat map representation of the DEGs between control and IF patients across different microarrays identified from the meta-analysis. Each color above represents a single dataset. The heat map was rescaled to prevent domination by study-specific effects. (**B**) Unsupervised clustering of the transcriptome of the DEGs in the three datasets.

Meta-analysis using a rank product method identified a total of 182 genes consistently differentially expressed in IF group compared with controls across three microarray datasets (pfp < 0.05). Among the 182 candidate implantation-associated genes, 119 genes were up-regulated and 63 were down-regulated in IF patients compared with controls. The complete list of DEs is provided in Supplementary Table 1. The down-regulated gene with the lowest pfp (average FC = 10.13, pfp = 3.97 × 10^–13^ ) was *PAEP* (progestagen associated endometrial protein), which is known to play an important role in regulating the endometrial environment suitable for implantation via immunomodulatory mechanisms [21]. A heat map visualization of the DEs across the three datasets is displayed in Figure 2A. To ascertain whether the expression profiles of DEs can distinguish the IF from control patient, we performed the unsupervised hierarchical clustering. Overall, the same sample type clustered together, with the exception that three IF samples clustered in control group and one control sample in IF group (Figure 2B).

### Functional analysis

**Figure 3:**
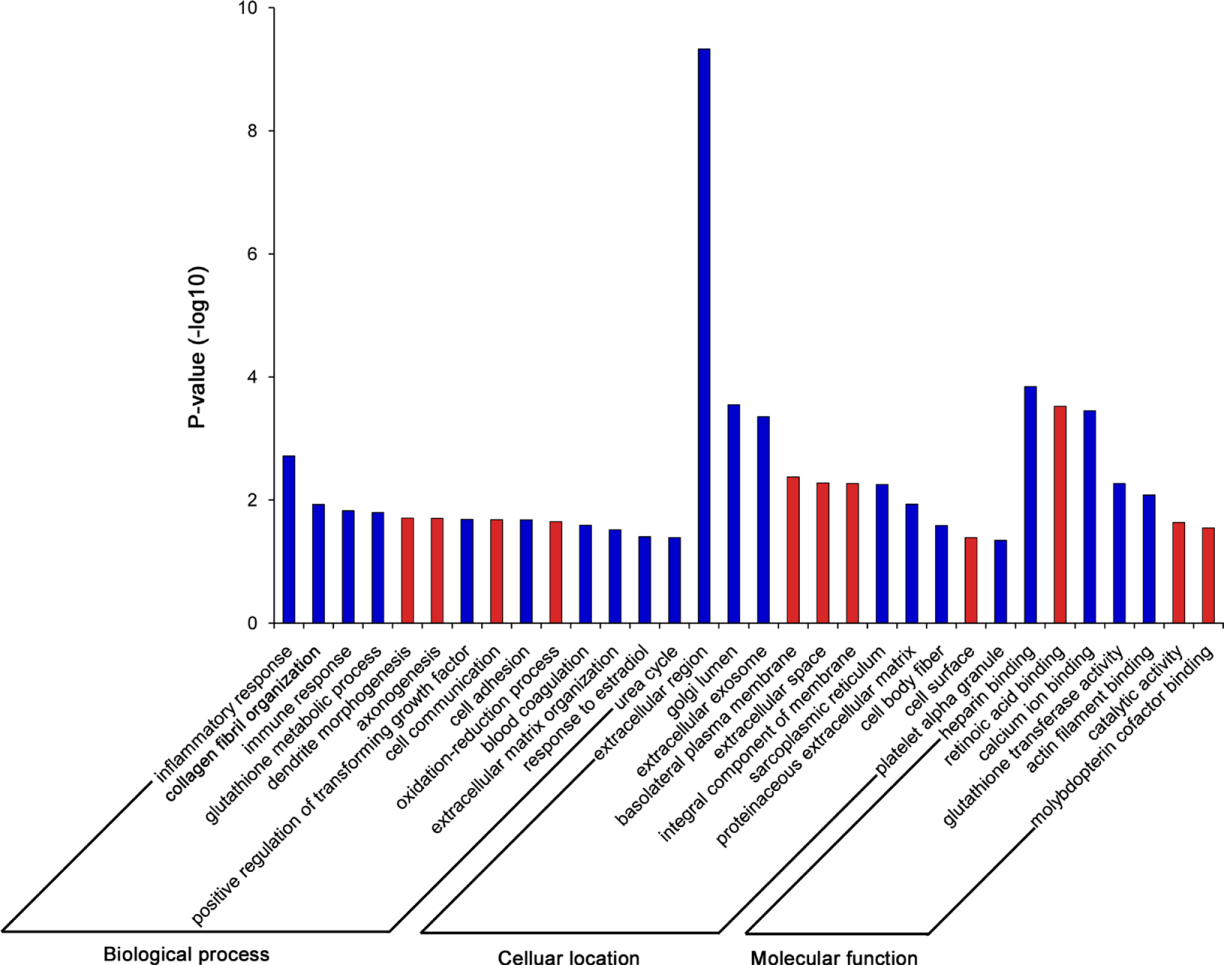
GO enrichment analysis for the up- and down-regulated genes

To identify biological processes associated with gene expression differences in control vs. IF patient, we performed GO enrichment analysis with the up- and down-regulated genes separately. Interestingly, there were many more enriched GO terms with down-regulated genes (66%, Figure 3), indicating that the down-regulation of genes in endometrium might be the underlying main cause for implantation failure. Therefore we focused on the down-regulated genes and found that GO terms for biological process significantly enriched in inflammatory (GO:0006954, *P* = 1.91 × 10^–3^) and immune response (GO: 0006955, *P* = 1.49 × 10^–2^), while for molecular functions, the enriched GO terms were heparin binding (GO:0004364, *P* = 1.43 × 10^–4^) and calcium ion binding (GO:0005509, *P* = 3.54 × 10^–4^), and for cellular component, the enriched GO terms were extracellular region (GO:0005576, *P* = 4.67×10^–10^) and golgi lumen (GO:0005796, *P* = 2.81×10^–4^). The processes of embryo implantation are considered analogous to inflammatory and immune responses [9, 16, 22, 23], thus we speculated that these down-regulation genes involved in inflammatory and immune responses might play important roles in regulating the endometrial receptivity for implantation.

### Network analysis

**Figure 4:**
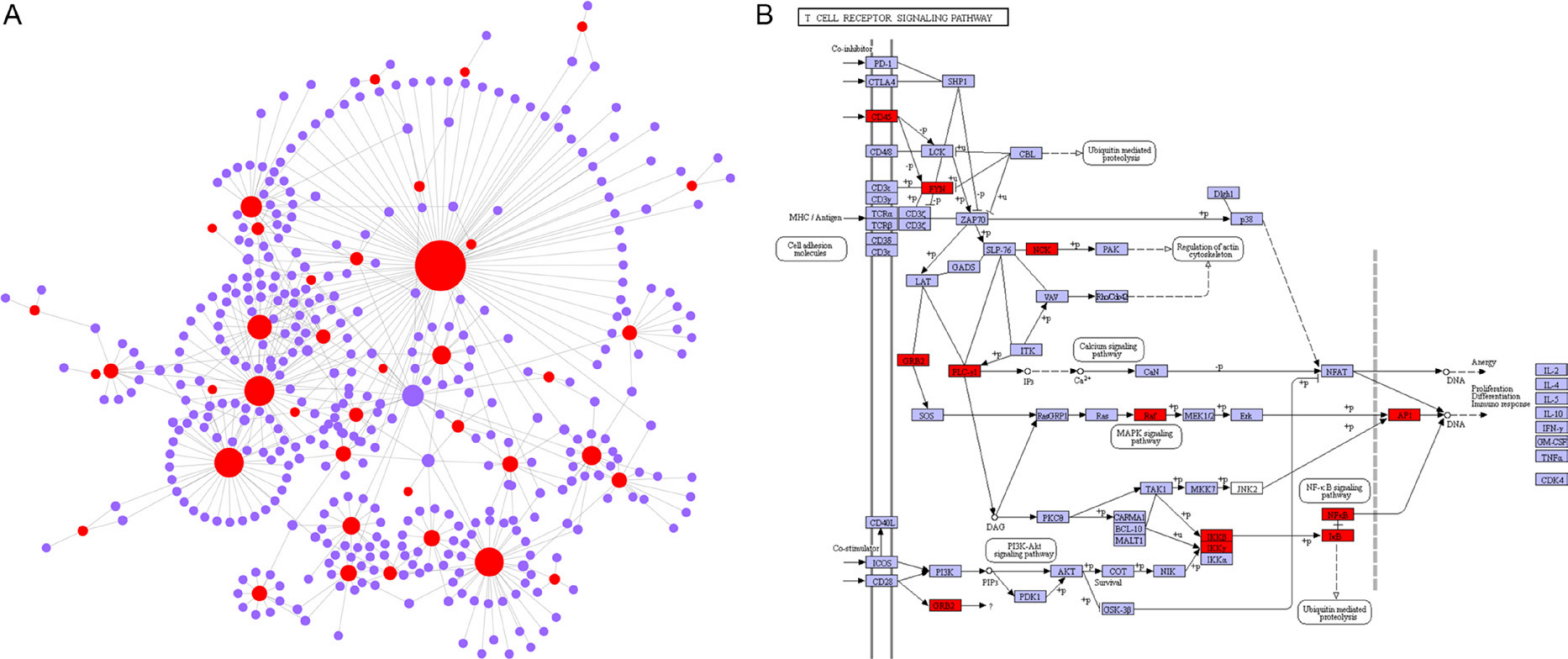
The network analysis of down-regulated genes in endometrium of IF patients (**A**) Network including 434 nodes and 484 edges. *Red*: down-regulated genes. *Purple*: interaction genes. (**B**) The network genes are enriched in T cell receptor signaling pathway. Red rectangles represent the genes in the network. Purple is the color of Kyoto Encyclopedia of Genes and Genomes (KEGG) database.

To further predict the function of these down-regulated genes in IF patients, a network analysis was performed. The resulting network contained 434 nodes and 484 edges (Figure 4A). The most highly ranked genes containing *GADD45A* (growth arrest and DNA damage inducible alpha, Degree = 77), *GZMB* (granzyme B, Degree = 38) and *NLRP2* (NLR family pyrin domain containing 2, Degree = 37) (Supplementary Table 2). To investigate the functions of the 434 genes in the network, we mapped them to the Kyoto Encyclopedia of Genes (KEGG) database. Hypergeometric test with *P* value < 0.05 was used as the criteria for pathway detection (Supplementary Table 3). The results showed that majority of the enrichments were inflammation and immune related pathways, for instance, the T cell receptor signaling pathway (Figure 4B). In addition, we also identified cell cycle pathyway, indicative of a reduced rate of cellular proliferation in endometrium. This is consistent with previous studies of IF that also reported cell cycle enrichment for the down-regulated genes [12].

### Validation of the expression of *NLRP2*

**Figure 5:**
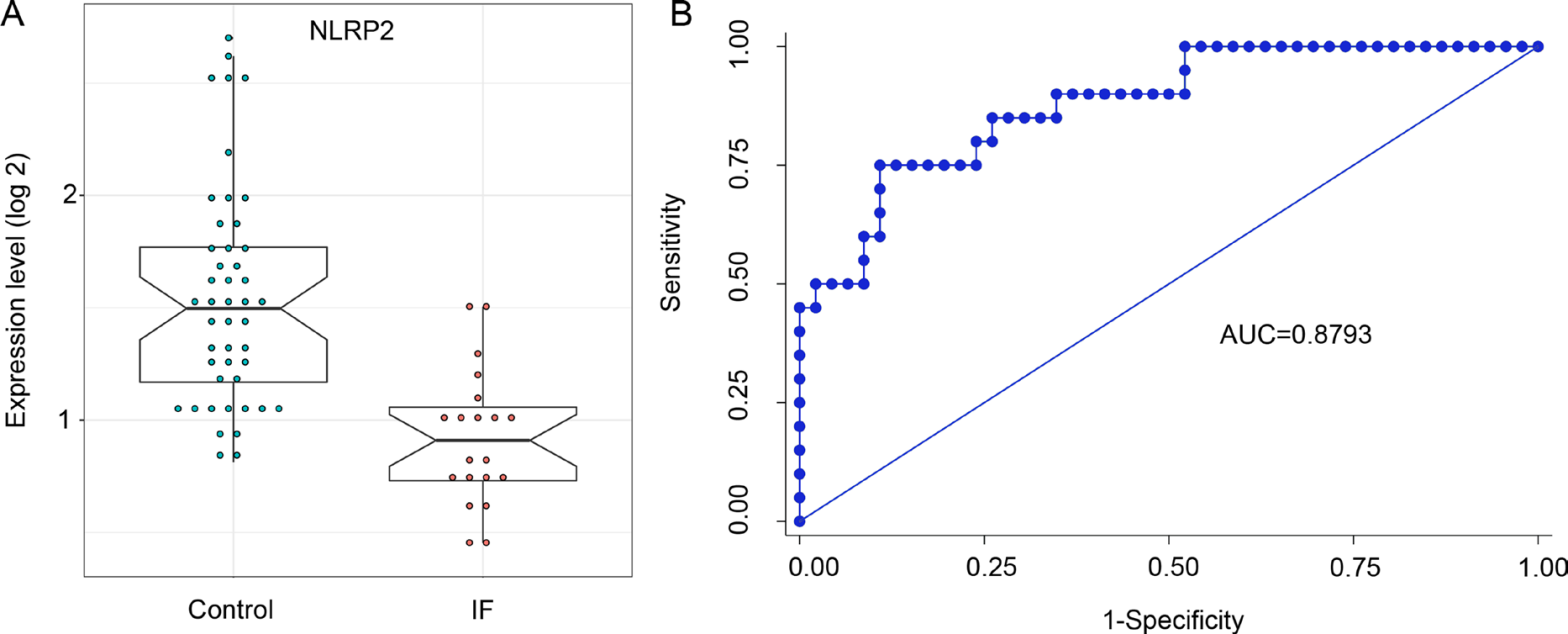
Evaluation of NLRP2 as predictive biomarker for clinical pregnancy in IVF (**A**) Relative abundance of *NLRP2* in endometrium of IF patients (red circles) compared with control (green circles) in samples from the datasets of GSE58144. A Student T test (two-tailed) was used to estimate the significance between IF and control patients. (**B**) ROC curves analysis for clinical pregnancy prediction by NLRP2 expression in endometrium.

The previous reports have suggested that controlled inflammation and activation of the immune response is essential for embryo implantation. Thus, we selected *NLPR2*, involved in inflammatory responses with the most degree in the network, for the validation of different expression. Here, we performed the expression quantification analysis for *NLPR2* in endometrium between IF and control patients using another non-GPL570 microarray data. The expression results revealed that *NLPR2* was significantly down-regulated in IF patients (Figure 5A). Furthermore, ROC analysis of *NLRP2* was performed to evaluate the diagnostic accuracy. The area under the curve was 87.93%, and the sensitivity and specificity reached 60.0% and 91.3% (Figure 5B).

## DISCUSSION

Many genes have been identified as molecular biomarkers of endometrial receptivity using high-throughput technology [12, 15–19]. However, the large number of biomarkers and the lack of explanation about their interactions make it extremely difficult to display a comprehensive overview of the process in a holistic way. Therefore, it is extremely important to identify the consistent biomarkers of endometrial receptivity. A meta-analysis that includes massive amounts of data from publicly available transcriptomes will be more accurate and improve biological understanding compared with individual analysis [20, 24]. Here, we employed a meta-analysis strategy on combined DEGs that were collected from different origins to highlight genes that were consistently differentially expressed with statistical significance between IF and control patients.

An appropriate analysis strategy and statistical methodology is crucial for the meta-analysis. Here, we normalized these expression datasets separately for addressing heterogeneity. In this condition, any measurement errors from a single data would have no influence on the final results with our normalization strategy [25]. For statistical analysis, we applied the nonparametric rank product method because it outperforms the other methods in terms of sensitivity and specificity, especially among multiple datasets [20, 26, 27]. Overall, our analysis strategy properly reduced the effect of heterogeneity derived from the different datasets and provided control over the extent to which false positives were included.

In total, we identified 182 genes that were differentially expressed in IF patients compared to control, including 119 up-regulated and 63 down-regulated genes. Using the expression patterns of these DEGs, we successfully distinguished the IF from the control patients through unsupervised hierarchical clustering, suggesting the potential of the DEGs to be the biomarkers of endometrial receptivity. An important gene *PAEP* was identified as the most down-regulated in our meta-analysis. Progesterone secreted by corpus luteum stimulates the expression of *PAEP* in WOI. It is also reported to have high expression in endometrium and was down regulated in IF patients undergoing IVF cycle [28–31]. Therefore, the down-regulation of *PAEP* leads to the endometrial deregulation in IF patients.

GO enrichment analysis displayed a strong skew towards down-regulation of processes in IF patients. This is consistent with previous studies of IF that reported enrichment for inflammatory and immune response within the set of down-regulated genes [16]. In addition to this, the KEGG pathyway analysis of network also showed the dysregulated pathyways, including T cell and B cell receptor signaling pathway (Figure 4B), Natural killer cell pathway and myeloid leukemia, indicating the down-regulated function of positive regulation of inflammatory and immune response during WOI. Rencent studies have demonstrated that the down-regulation of inflammation might lead to the impaired elicitation of immune cells as well as their recruitment to the endometrium [32, 33]. Thus, this would be the main cause of defect in creating suitable environment in endometrium during WOI leading to IF. Furthermore, we detected the down-regulation in IF patients of genes involved in cell cycle and apoptosis regulation (Supplementary Table 3), indicative of a reduced rate of cell proliferation in endometrium. Additionally, in this meta-analysis, the down-regulation genes in IF patients were also enriched in estradiol response, chemokines, growth factors, which have been found to be important for the maintenance of microenvironment during implantation supporting the endometrium-embryo crosstalk [34, 35].

Based on network analysis, *NLPR2* was identified as the most significant hub gene in inflammatory pathway. NLRP family are important regulatory factors of innate immunity and inflammation [36, 37]. Interestingly, maternal deficiency of NLRP2 can cause embryonic lethality in mice [38, 39], suggesting its vital roles in reproduction. However, till data there is no reports about the association between *NLRP2* and endometrial receptivity. A biomarker-based diagnose for endometrial receptivity could have great impact in clinical scenario. Here, the evaluation of biomarker performance showed that *NLRP2* can distinguish IF patients from controls with 60.0% sensitivity and 91.3% specificity. However, validation of the biomarker in prospective cohorts of IVF patients would be required.

In summary, we performed a meta-analysis of endometrial gene expression and identified a list of important gene signature that might contribute to the endometrial deregulation in IF patients. In addition, our results strengthen the association between endometrial receptivity and several pathyways, especially the inflammatory and immune pathways, and provide insights into the molecular mechanisms underlying the impairment of inflammatory and immune signaling observed in IF patients. Furthermore, this study underscores the potential of network analysis as a powerful framework to gain insight into the mechanisms underlying endometrial receptivity and to identify potential biomarkers. Further functional studies and validated test about the role of these biomarkers in endometrial receptivity are warranted.

## MATERIALS AND METHODS

### Identification of eligible gene expression dataset

To compare the difference of human endometrium derived from control and IF patients in IVF cycle, the NCBI GEO (Gene Expression Omnibus) database (http://www.ncbi.nlm.nih.gov/geo/) was searched for eligible expression datasets using the key words ‘endometrium’ and ‘microarray’ on June 1, 2017. Datasets that met the following inclusion criteria were included: 1) all two types of endometrium tissue (IF and control) were contained in one experiment; 2) GeneChip Human Genome U133 Plus 2.0 arrays GPL570 platform; 3) there were at least three replicates in each sample. Data were extracted from the original studies by two independent reviewers. Any discrepancies between reviewers were resolved by consensus or a third reviewer. The microarray information of the GPL15789 microarray platform for results validation in this study was obtained from GSE58144.

### Meta-analysis of microarray datasets

A total of three microarray datasets passed the inclusion criteria and were considered for subsequent analysis. The datasets analyzed in this study are listed in Table 1. For the microarray analyses, the raw data were log2-transformed and quantile normalized independently for individual datasets using RMA in R (Bioconductor). When multiple probes were mapped to the same gene, those probes were averaged. To avoid bias from the heterogeneity among multiple datasets, we used a non-parametric approach based on rank order in the RankProd package to detect differentially expressed genes (DEs) that were consistently highly ranked in different datasets. Briefly, this method combines the gene rank from different datasets together to select the DE. The fold changes (FCs) are computed for all possible pairwise comparisons. The ranks of the FCs within each comparison are then used to calculate the rank product foreach gene. We used 1000 permutations to obtain the percentage of false positive predictions (pfp), which is also known as the false discovery rate (FDR). As recommended, a pfp value of 0.05 was used to set the threshold for DEs.

### Function enrichment analysis

The Database for Annotation, Visualization and Integrated Discovery (DAVID) was a frequently-used bioinformatics resources for GO functional annotation. First, we upload gene lists to DAVID. And then, after selecting identifier for thes genes (In this work, we select “OFFICIAL_GENE_SYMBOL). Biological process, molecular fuction and cellular component terms was seleted as background gene sets respectively. Hypergeometric Exact test was used to measure gene-enrichment in background annotation terms.

### Network analysis

We used a comprehensive high-quality PPI database downloaded from the InnateDB [30], which participates in the International Molecular Exchange (IMEx) consortium [31]. The database was derived by manually curating protein interaction data from published literature and by integrating experimental data from several PPI databases including MINT [32], IntAct [33], BIND, BioGRID [34] and DIP [35]. The gene networks were visualized using Cytoscape 3.2.0 [36].

### Statistical analysis

Statistical analysis was performed using STATA12.0 software (Statacorp, TX, USA). The Student *t* test (two-tailed) was used to estimate the significance between IF and control patients for numerical variables. Receiver operating characteristic curves (ROC) were used to evaluate the prognostic ability of biomarker to successful pregnancy. The area under the ROC curve and the sensitivity and specificity were also calculated.

## SUPPLEMENTARY MATERIALS TABLES









## Abbreviations

IF: implantation failure
ART: assisted reproductive techniques
IVF: *in vitro* fertilization (IVF)
DEGs: differentially expressed genes
