# Supplementary material for: Meta-analysis identifies candidate key genes in endometrium as predictive biomarkers for clinical pregnancy in IVF
Source: Oncotarget. 2017 Oct 26;8(60):102428–36. doi: 10.18632/oncotarget.22096 (PMC5731968; doi:10.18632/oncotarget.22096)
Supplement: Supplementary file 1 [file oncotarget-08-102428-s001.pdf]

Meta-analysis identifies candidate key genes in endometrium as predictive biomarkers for clinical pregnancy in IVF

SUPPLEMENTARY MATERIALS

Supplementary Table 1: The detail results of DEGs. See Supplementary_Table_1

Supplementary Table 2: The degree results of these genes in network. See Supplementary_Table_2

Supplementary Table 3: The KEGG enrichment analysis of these genes in network. See Supplementary_Table_3
